# Supplementary material for: Is [177Lu]Lu-PSMA-617 Redefining Value in mCRPC Care? A Meta-Analysis of Clinical and Economic Endpoints
Source: Cancers (Basel). 2025 Jul 4;17(13):2247. doi: 10.3390/cancers17132247 (PMC12248729; doi:10.3390/cancers17132247)
Supplement: Supplementary file 1 [file cancers-17-02247-s001.zip › Tabella S1.pdf]

Supplementary Table S1: The CHEERS 2022 Checklist for economic evaluation

CHEERS Item	Summary
Title	Clearly identifies the study as an economic evaluation. The title reflects the integration of clinical and economic outcomes.
Abstract	Structured abstract includes background, methods, results (both clinical and economic), and conclusions.
Background and objectives	States the clinical need in mCRPC and the purpose of evaluating [177Lu]Lu-PSMA-617 in terms of both efficacy and economic impact.
Target population and subgroups	Patients with metastatic castration-resistant prostate cancer (mCRPC) treated in randomized trials.
Setting and location	Multi-center, multinational RCTs across Europe, North America, and Australia.
Study perspective	Health system perspective; only direct treatment-related costs were considered.
Comparators	Standard of Care (SoC), ARTA, or Cabazitaxel as defined in each included trial.
Time horizon	Aligned with the duration of follow-up in clinical trials; no long-term modeling was performed.
Discount rate	Not applicable, as long-term projections and utilities were not estimated.
Choice of health outcomes	Overall survival (OS) and radiographic progression-free survival (rPFS) as reported in source RCTs.
Measurement of effectiveness	Hazard ratios and survival probabilities extracted from Kaplan–Meier curves using validated digitization and meta-analytic techniques.
Measurement and valuation of preference-based outcomes	Not applicable; utility scores and QALY estimates were not collected or modeled.
Estimating resources and costs	Costs for Lu-PSMA-617 and comparators (Cabazitaxel, ARTA, SoC) obtained from literature and trial protocols.
Currency, price date, and conversion	Costs standardized to 2024 euros using published national health system data.
Rationale and description of model	No formal model (e.g., Markov) was implemented. A simplified ICER approach was used with cost per rPFS month gained.

Analytics and assumptions	ICER calculated from treatment cost and rPFS AUC. Assumes treatment stops at progression.
Characterising heterogeneity	Population characteristics (e.g., visceral metastases, PSA, ALP) compared across studies to assess baseline imbalances.
Characterising uncertainty	No probabilistic sensitivity analysis; heterogeneity and baseline imbalance addressed qualitatively.
Characterising distributional effects	Not assessed.
Discussion	Addresses limitations of the simplified economic approach and explains clinical and economic findings in light of baseline population features.
Source of funding	No industry funding; institutional academic analysis.
Conflicts of interest	None declared.
