# Supplementary material for: Is [177Lu]Lu-PSMA-617 Redefining Value in mCRPC Care? A Meta-Analysis of Clinical and Economic Endpoints
Source: Cancers (Basel). 2025 Jul 4;17(13):2247. doi: 10.3390/cancers17132247 (PMC12248729; doi:10.3390/cancers17132247)
Supplement: Supplementary file 1 [file cancers-17-02247-s001.zip › Tabella S2.pdf]

Supplementary Table S2: Study-Level Heterogeneity Across Included Trials

Study	Study Design & Phase	Prior Lines of Therapy	Comparator Arm	Inclusion Criteria (PSMA PET)	ECOG (0–1)	% Visceral Mets	Median PSA (ng/mL)	ARTA Exposure	Comments
TheraP	RCT, Phase 2	1–2 prior therapies	Cabazitaxel	≥20 SUVmax at 1 site, >10 elsewhere	97%	21% (Lung+Liver)	94	Post-ARTA	Strict PET-based inclusion
VISION	RCT, Phase 3	Heterogeneous	SOC (ARTA ± chemo)	≥1 PSMA+ lesion, no PSMA– lesions	95%	21.3%	93.2	Post-ARTA	Open-label, multicenter
ENZA-p	RCT, Phase 2	Mostly pre-chemo	Enzalutamide continuation	≥15 at 1 site, >10 all sites	n.r.	8%	39 (IQR 13–75)	Concurrent	Maintenance strategy post-PSMA
PSMAfore	RCT, Phase 3	ARPI-naïve (post-ADT)	ARPI	≥1 PSMA+ lesion, no PSMA– lesions	0–2	15%	n.r.	Pre-ARTA	Early use of Lu-PSMA
SPLASH	RCT, Phase 3	Mixed	ARPI	PSMA+ centrally confirmed, SUV not reported	~99%	n.r.	13.2 (Lu), 18.9 (ARPI)	Mixed	10.1% patients were M0 at inclusion
