# Supplementary figures and images for: Is [177Lu]Lu-PSMA-617 Redefining Value in mCRPC Care? A Meta-Analysis of Clinical and Economic Endpoints
Source: Cancers (Basel). 2025 Jul 4;17(13):2247. doi: 10.3390/cancers17132247 (PMC12248729; doi:10.3390/cancers17132247)

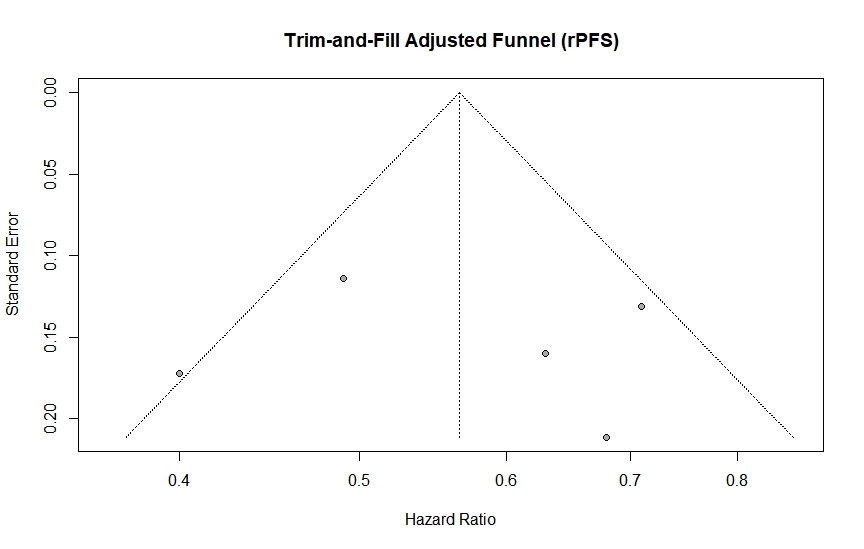

Supplement: Supplementary file 1 [file cancers-17-02247-s001.zip › figura S3 publlication bias.jpeg]

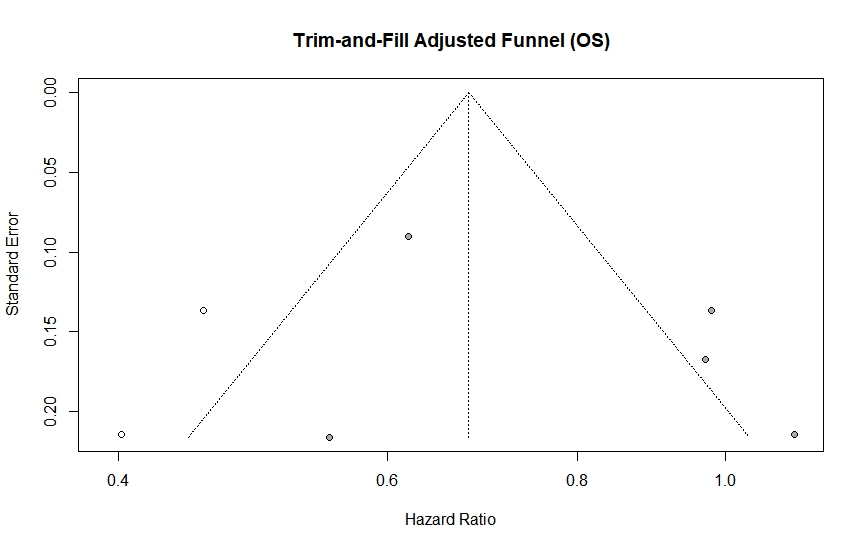

Supplement: Supplementary file 1 [file cancers-17-02247-s001.zip › figura S4 OS.jpeg]

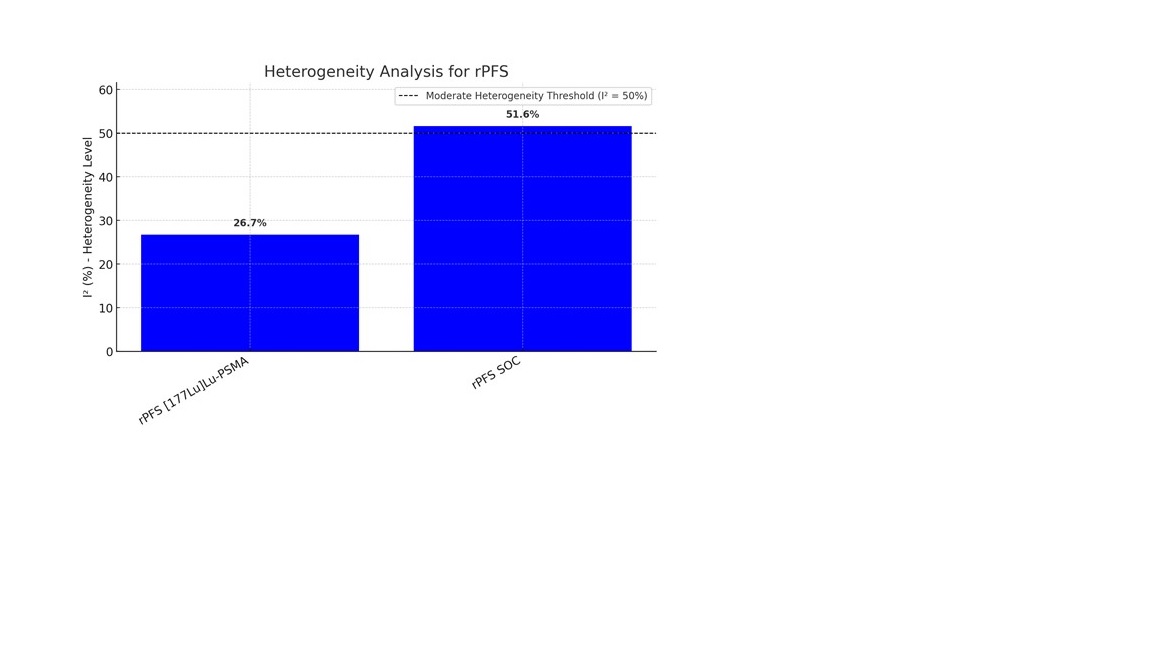

Supplement: Supplementary file 1 [file cancers-17-02247-s001.zip › figure S1.jpg]

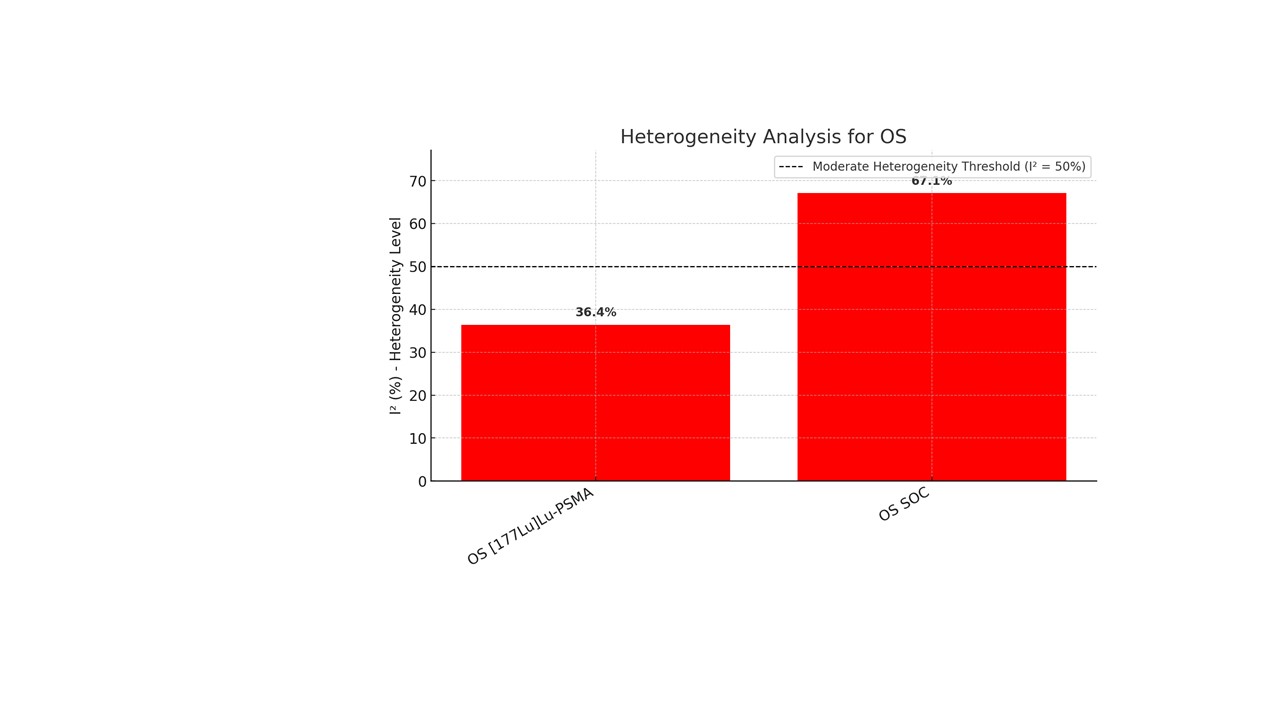

Supplement: Supplementary file 1 [file cancers-17-02247-s001.zip › figure S2.jpg]
